# Determinants of mortality in non-neutropenic ICU patients with candidaemia

**DOI:** 10.1186/cc7964

**Published:** 2009-07-13

**Authors:** Deborah JE Marriott, E Geoffrey Playford, Sharon Chen, Monica Slavin, Quoc Nguyen, David Ellis, Tania C Sorrell

**Affiliations:** 1 St Vincent's Hospital, Victoria Street, Darlinghurst, NSW 2010, Australia; 2 University of New South Wales, Kensington, Sydney, NSW 2052, Australia; 3 Princess Alexandra Hospital, Ipswich Road, Woolloongabba, Qld 4102, Australia; 4 University of Sydney, Camperdown, Sydney, NSW 2006, Australia; 5 Westmead Hospital, Darcy Road, Westmead, NSW 4152, Australia; 6 Royal Melbourne Hospital, Grattan Street, Parkville, Vic 3050, Australia; 7 Women's and Children's Hospital, King William Road, Adelaide, SA 5006, Australia

## Abstract

**Introduction:**

Candidaemia in critically-ill intensive care unit (ICU) patients is associated with high crude mortality. Determinants of mortality – particularly those amenable to potential modification – are incompletely defined.

**Methods:**

A nationwide prospective clinical and microbiological cohort study of all episodes of ICU-acquired candidaemia occurring in non-neutropenic adults was undertaken in Australian ICUs between 2001 and 2004. Multivariate Cox regression analyses were performed to determine independently significant variables associated with mortality.

**Results:**

183 episodes of ICU-acquired candidaemia occurred in 183 patients during the study period. Of the 179 with microbiological data, *Candida albicans *accounted for 111 (62%) episodes and *Candida glabrata*, 32 (18%). Outcome data were available for 173: crude hospital mortality at 30 days was 56%. Host factors (older age, ICU admission diagnosis, mechanical ventilation and ICU admission diagnosis) and failure to receive systemic antifungal therapy were significantly associated with mortality on multivariate analysis. Among the subset who received initial fluconazole therapy (n = 93), the crude mortality was 52%. Host factors (increasing age and haemodialysis receipt), but not organism- (*Candida *species, fluconazole MIC), pharmacokinetic- (fluconazole dose, time to initiation), or pharmacodynamic-related parameters (fluconazole dose:MIC ratio) were associated with mortality. Process of care measures advocated in recent guidelines were implemented inconsistently: follow-up blood cultures were obtained in 68% of patients, central venous catheters removed within five days in 80% and ophthalmological examination performed in 36%.

**Conclusions:**

Crude mortality remains high in Australian ICU patients with candidaemia and is overwhelmingly related to host factors but not treatment variables (the time to initiation of antifungals or fluconazole pharmacokinetic and pharmacodynamic factors). The role and timing of early antifungal intervention in critically-ill ICU patients requires further investigation.

## Introduction

Candidaemia is a relatively common healthcare-associated infection in critically-ill patients in intensive care units (ICUs) [1-3] that is associated with poor clinical outcomes and excess economic costs [4,5].

Despite the availability of new antifungal agents and management guidelines [6], candidaemia remains associated with persistently high crude mortality rates. Interest has therefore centred on potentially modifiable treatment-related outcome determinants. In particular, improved outcomes among predominantly non-ICU patient cohorts have been associated with earlier initiation of antifungal therapy and for fluconazole regimens optimised for pharmacodynamic parameters [7-10]. However, the generalisability of these findings to critically-ill ICU patients remains unknown. We therefore assessed the association of outcome with host-, microbial-, and treatment-related factors among a large prospective Australia-wide cohort of ICU patients with candidaemia. Although the overall population-based epidemiology of candidaemia in Australia has been previously reported as part of the Australian Candidaemia Study [11], episodes specifically occurring in adult non-neutropenic ICU patients have now been analyzed and presented here to describe their outcomes and prognostic factors.

## Materials and methods

### Study design

The Australian Candidaemia Study involved a three-year prospective nationwide surveillance of all episodes of candidaemia in Australia from August 2001 to July 2004 as reported elsewhere [11]. Fifty of 52 Australian public and private microbiology laboratories participated in the study. Clinical information on each episode was collected on a standardised data form at day 5 and day 30 following the first isolation of *Candida *species from blood. Data included patient demographics, major concomitant conditions, risk factors occurring within the preceding 30 days (such as surgical and other invasive interventions, vascular access devices, and receipt of total parenteral nutrition, haemodialysis, immunosuppressive therapies and antimicrobial agents), source of candidaemia, clinical signs of sepsis, complications, results of diagnostic studies (including serum creatinine at days 1 and 5), antifungal therapy and clinical outcomes at 30 days. *Candida *isolates were forwarded to reference laboratories for phenotypic and genotypic species identification (performed in the National Mycology Reference Laboratory, Women's and Children's Hospital, Adelaide and the Molecular Mycology Reference Laboratory, Westmead Hospital, Sydney, respectively), and susceptibility testing using Clinical Standards Laboratory Institutes methodology [12] (performed in the National Mycology Reference Laboratory). Approval for the study was obtained from the Human Research Ethics Committees of all participating institutions. Informed written consent was obtained from patients that were included in the study.

### Definitions

The definition of ICU acquisition of candidaemia was the occurrence of the first positive blood culture growing *Candida *species at 48 hours or more following ICU admission or 48 hours or less following ICU discharge. Paediatric, neonatal or neutropenic (absolute neutrophil count ≤ 1 × 10^9 ^neutrophils/L) patients were excluded. ICUs included hospital wards or units providing invasive ventilatory and/or intensive haemodynamic support; high dependency units and coronary care units were excluded. Risk factors over the 30 days prior to onset of candidaemia were assessed. Vascular access device-related candidaemia required the isolation of the same *Candida *species from both blood and catheter tip. Relapses were defined as recurrent positive blood cultures with the same species within 30 days of the original positive blood culture after an initial clinical and microbiological response.

### Statistical analyses

Clinical data were analyzed using SPSS (Version 16.0, SPSS, Chicago, IL, USA). Incidences were calculated using ICU admission data from participating ICUs covering the study period. Univariate analyses were performed using the Student's t test (continuous variables) and chi-squared or Fisher's exact tests (categorical variables). Assessment of factors associated with mortality were performed using multivariate Cox regression models with hospital mortality as the dependant variable, censored for hospital discharge, using the backwards selection method after initially including all biologically-plausible variables, and those with an unadjusted association of *P *< 0.2. A *P *< 0.05 was set as the limit for acceptance or removal of variables. For all analyses, the fluconazole dose was adjusted for renal impairment [13] and the fluconazole minimum inhibitory concentration (MIC) and fluconazole dose:MIC ratio were log_10 _transformed. Survival analyses were performed on two separate patient cohorts: the entire ICU cohort and the subset of patients in whom fluconazole was the sole initial antifungal therapy during the initial 72 hours of therapy (but exclusion of patients with breakthrough infection, defined as occurrence of candidaemia more than 72 hours prior to collection of the first positive blood culture).

## Results

### Cases and incidence of candidaemia in ICU

Over the three-year study period, there were 183 episodes of ICU-acquired candidaemia in 183 patients from 38 ICUs. The mean age ± standard deviation was 58.6 ± 18.6 years and 57% were male. Most patients had undergone a recent surgical procedure (67%), had received recent antimicrobial therapy (97%) and were ventilated at the time of candidaemia diagnosis (79%). The median time from ICU admission to development of candidaemia was eight days (interquartile range (IQR), 5 to 15 days; range, 2 to 86 days). Almost three-quarters of episodes (74%) occurred in tertiary-referral hospital ICUs. The overall incidence of ICU-acquired candidaemia calculated from 19 ICUs (of the 22 ICUs reporting at least three candidaemia episodes) ranged from 0.53 to 6.46 per 1000 ICU admissions (mean, 2.06 per 1000 admissions; 95% confidence interval, 1.73 to 2.44).

### Species distribution and antifungal susceptibilities

*Candida albicans *accounted for 62% (111/178) of episodes, *Candida glabrata *for 18% (32), *Candida parapsilosis *for 8% (14), *Candida tropicalis *for 6% (10), *Candida krusei *for 4% (7), *Candida dubliniensis *for 1% (2), and other *Candida *species accounted for 3% (5) of episodes. There were two mixed infections (one *C. albicans*/*C. glabrata *and one *C. glabrata*/unidentified *Candida *species). Antifungal susceptibility results were available for 174 isolates (Table 1). Based on Clinical Standards Laboratory Institutes MIC breakpoints [14-16], all isolates were susceptible to amphotericin B, 167 (96%) to flucytosine, 136 (78%) to fluconazole, 115 (66%) to itraconazole and 172 (99%) to voriconazole. Of the 32 *C. glabrata *isolates, two (6%) were susceptible to fluconazole, 23 (72%) were susceptible-dose dependent and seven (22%) were resistant. Two of the fluconazole-resistant isolates were also resistant to voriconazole. All seven *C. krusei *isolates were susceptible to voriconazole. All of the other 135 *Candida *isolates were fluconazole susceptible with the exception of one isolate of *C. albicans *that demonstrated dose-dependent susceptibility. The MIC_90 _for caspofungin was 0.25 μg/mL (n = 54: Table 1).

**Table 1:** *In vitro *antifungal susceptibility of *Candida *species isolated from ICU-acquired candidaemia episodes

Agent	MIC*	*Candida albicans *(n = 106)	*Candida glabrata *(n = 32)	*Candida krusei *(n = 7)	*Candida parapsilosis *(n = 14)	*Candida tropicalis *(n = 10)
AMB	MIC_50_	0.125	0.25	0.25	0.5	0.5
	MIC_90_	0.25	0.5	0.5	0.5	0.5
5FC	MIC_50_	0.125	0.03	8	0.125	0.06
	MIC_90_	0.5	0.03	16	0.25	0.125
FLU	MIC_50_	1	32	64	2	1
	MIC_90_	2	64	>64	4	4
ITR	MIC_50_	0.06	2	0.5	0.25	0.25
	MIC_90_	0.125	16	0.5	0.5	0.5
VOR	MIC_50_	0.016	0.5	0.5	0.06	0.06
	MIC_90_	0.03	1	0.5	0.25	0.5
POS	MIC range (no. tested)	0.008 to 0.06 (16)	0.03 to 8 (7)	0.125 (1)	-	0.008 to 0.06 (4)
CAS	MIC range (no. tested)	0.03 to 0.25 (27)	0.06 to 0.25 (11)	0.25 to 1 (3)	0.125 (1)	0.06 to 0.5 (7)

AMB = amphotericin B; CAS = caspofungin; 5FC = 5-flucytosine; FLU = fluconazole; ITR = itraconazole; POS = posaconazole; MIC = minimum inhibitory concentration (μg/mL); VOR = voriconazole.

### Clinical characteristics, complications and management of candidaemia

Manifestations of sepsis [17] were common both at diagnosis of candidaemia (84%) and at day 5 (76%). The source of candidaemia was attributed to an intravascular device in 35%, an intra-abdominal source in 10%, the urinary tract in 3%, other sources in 5% and an unknown source in 47%.

Ophthalmological manifestations consistent with intraocular candidiasis were demonstrated in six of 48 (13%) patients who underwent ocular examination. Other infective complications of candidaemia included nine episodes of metastatic renal infections, three cases of endocarditis (all graded 'possible' infection by Duke's criteria [18]) and two patients with hepatosplenic candidiasis (documented by computed tomography scan and post-mortem examination). Relapses occurred in 24 of 183 (13%) episodes. Neither metastatic infective foci nor relapses were associated with specific *Candida *species or any underlying co-morbidity.

Antifungal therapy was initiated in 156 (85%) patients: of these, fluconazole in 76%, amphotericin B deoxycholate in 12%, a lipid formulation of amphotericin B in 4%, caspofungin in 4%, and voriconazole or posaconazole in 3%. There was considerable variation in the time to initiation of antifungal therapy: in 21% it was initiated within 24 hours of drawing the first positive blood culture, in 14% between 24 and 48 hours, in 29% between 48 and 72 hours, and in 35% greater than 72 hours.

Other processes of care were also assessed. Among patients surviving five days, follow-up blood cultures were obtained in 68% and central venous catheters removed within five days in 80%. Among patients surviving 30 days, an ocular examination had been performed in 36% (90% of which were performed by an ophthalmologist).

### Determinants of mortality

Among the entire ICU cohort with outcome data (n = 173), the crude in-hospital 30-day mortality was 56%, with median time to death after drawing the first positive blood culture of seven days (IQR, 2 to 12 days). Variables associated with an increased risk of death by multivariate Cox regression analyses are presented in Table 2: those independently associated by multivariate analysis included host-related factors (increasing age, mechanical ventilation at time of candidaemia and management in the ICU for reasons other than multitrauma) and non-receipt of systemic antifungal therapy. Several other host-related factors (total parenteral nutrition, receipt, haemodialysis receipt and presence/non-removal of vascular access devices) were also associated with mortality on univariate – but not multivariate – analysis. Of note was the lack of association with mortality for different *Candida *species or for delays in initiation of antifungal therapy.

**Table 2:** Risk factors for hospital mortality on entire ICU cohort with candidaemia

Variable	Dying patients*	Surviving patients*	Univariate analysis**		Multivariate analysis†	
				
			Unadjusted HR (95% CI)	*P*	Adjusted HR (95% CI)††	*P*
Male sex	55/97 (57%)	45/76 (59%)	1.00 (0.67 to 1.49)	0.99		
Age	63.3 ± 16.7 years	51.1 ± 19.2 years	1.03 (1.01 to 1.04)	<0.001	1.03 (1.01 to 1.4)	<0.001
Antifungal agents prior to diagnosis	10/96 (10%)	6/76 (8%)	1.03 (0.53 to 1.98)	0.93		
Non-receipt of antifungal agents after diagnosis	20/97 (21%)	5/76 (7%)	5.17 (3.08 to 8.68)	<0.001	7.90 (3.73 to 16.71)	<0.001
*Candida albicans*	43/97 (44%)	26/76 (34%)	0.73 (0.49 to 1.10)	0.13		
Vascular access device removed or not in place	55/80 (69%)	64/79 (84%)	0.41 (0.26 to 0.67)	<0.001		
TPN receipt	53/96 (55%)	26/76 (34%)	1.52 (1.02 to 2.28)	0.04		
Haemodialysis	23/97 (24%)	8/76 (11%)	1.66 (1.04 to 2.66)	0.03		
Corticosteroid receipt	33/97 (34%)	20/76 (26%)	1.36 (0.89 to 2.07)	0.17		
Non-multitrauma patient	93/97 (96%)	60/76 (79%)	3.25 (1.19 to 8.87)	0.02	6.97 (1.64 to 29.67)	0.009
Recent surgery	71/97 (73%)	47/76 (62%)	1.24 (0.79 to 1.94)	0.35		
Other healthcare related infections	73/97 (75%)	55/76 (72%)	0.85 (0.53 to 1.35)	0.49		
Ventilation at day 1	82/96 (85%)	55/76 (72%)	1.51 (0.86 to 2.67)	0.15	4.03 (1.93 to 8.41)	<0.001
Sepsis at day 1	86/97 (89%)	60/76 (79%)	1.33 (0.71 to 2.49)	0.37		
Time to initiation of systemic antifungal	2.0 ± 1.3 days	2.3 ± 1.6 days	0.88 (0.75 to 1.04)	0.13		

* n/N (%) or mean ± standard deviation shown; **Only significant (*P *< 0.05) and selected non-significant variables on univariate analysis are shown; †Only significant variables on multivariate analysis are shown; ††Adjusted for other variables in the model.

CI = confidence interval; HR = hazard ratio; ICU = intensive care unit; TPN = total parenteral nutrition.

Among the subset of patients who received initial fluconazole therapy for the first 72 hours (n = 93), crude mortality was 52%. Variables independently associated with increased risk of death by multivariate Cox regression analysis included increasing age and haemodialysis receipt (Table 3). Although there was a non-significant trend between time to initiation of fluconazole and mortality (Table 4), this was not significant by multivariate analysis. Organism-related (*Candida *species and fluconazole MIC), pharmacokinetic-related (renal-adjusted fluconazole dose) or pharmacodynamic-related (fluconazole dose:MIC ratio) factors were not associated with mortality.

**Table 3:** Risk factors for hospital mortality on ICU patients with candidaemia initially treated with fluconazole

Variable	Dying patients, n (%)	Surviving patients, n (%)	Univariate analysis*		Multivariate analysis**	
				
			Unadjusted HR (95% CI)	*P*	Adjusted HR (95% CI)	*P*
Age			1.03 (1.01 to 1.05)	0.001	1.03 (1.01 to 1.05)	0.002
Haemodialysis receipt	10/48 (21%)	4/45 (9%)	1.65 (0.82 to 3.32)	0.16	2.12 (1.03 to 4.35)	0.04
TPN receipt	30/48 (63%)	15/45 (33%)	1.90 (1.06 to 3.42)	0.03		
Non-multitrauma patient	47/48 (98%)	33/45 (73%)	9.35 (1.29 to 67.84)	0.03		
Ventilation day 1	40/48 (83%)	34/45 (76%)	1.28 (0.60 to 2.75)	0.52		
*Candida glabrata *or *Candida krusei*	11/48 (23%)	3/45 (7%)	2.31 (1.17 to 4.56)	0.02		
Time to fluconazole initiation	1.80 ± 1.29 days	2.51 ± 1.74 days	0.80 (0.65 to 0.98)	0.03		
Fluconazole MIC (log_10 _transformed)	0.24 ± 0.74	0.10 ± 0.68	1.31 (0.90 to 1.90)	0.16		
Fluconazole dose (log_10 _transformed)	2.66 ± 0.22	2.62 ± 0.21	1.89 (0.52 to 6.82)	0.33		
Fluconazole dose: MIC (log_10 _transformed)	2.33 ± 0.22	2.45 ± 0.72	0.77 (0.54 to 1.11)	0.16		

CI = confidence interval; HR = hazard ratio; ICU = intensive care unit; MIC = minimum inhibitory concentration; TPN = total parenteral nutrition.

**Table 4:** Relationship between time to initiation of fluconazole and mortality

Time to initiation of fluconazole following date initial positive blood culture	Hospital mortality, n/N (%)
0	8/15 (53%)
1 days	10/15 (67%)
2 days	15/25 (60%)
3 days	6/16 (38%)
≥4 days	5/16 (31%)

## Discussion

The serious consequences of candidaemia among critically-ill patients in the ICU [4,5] are apparent in this three-year nationwide study, with crude in-hospital 30-day mortality rates of 56% and a median time from candidaemia to death of seven days. To improve these poor outcomes, the identification of potentially modifiable determinants of mortality is an urgent priority. Recent observational studies on mixed ICU/non-ICU cohorts with candidaemia have reported associations between mortality and delays in initiation of antifungal therapy and fluconazole regimens not optimised for target pharmacodynamic parameters [7-10]. We thus sought to assess whether these – or other – potentially modifiable factors were associated with mortality among critically-ill ICU patients with candidaemia.

As expected, among the entire cohort of candidaemic ICU patients, multivariate survival analysis revealed that host-related variables (including age, non-multitrauma patients and ventilation) and failure to receive antifungal therapy were associated with mortality. More than one-quarter of deaths involved patients not treated with antifungals; more than two-thirds of whom died within 48 hours of candidaemia onset (i.e. prior to blood culture positivity). Failure to initiate early antifungal therapy clearly represents a potentially modifiable mortality risk factor, and in this regard, predictive models to prospectively identify patients at high risk of candidaemia [19,20] as a trigger for early antifungal intervention may improve outcomes. However, it was of considerable interest that among treated patients in our cohort, delays in the initiation of antifungal therapy were not associated with greater mortality. Given recent reports of such an association [8,9], and the Infectious Diseases Society of America (IDSA) [21] guidelines, which recommend initiation of antifungal therapy within 24 hours of diagnosis, our discrepant findings require further examination.

Several factors may be relevant. We measured time to antifungal initiation in 24-hour increments, which may therefore have concealed a beneficial effect of very early treatment, given the 12-hour window period defined by Morrell and colleagues for a mortality difference [9]. However, it should be noted that in the study by Morrell and colleagues, only nine patients actually received antifungal therapy within 12 hours, and that across all other time periods, no progressive mortality increase was evident. In contrast, the other relevant study [8] did demonstrate increases in mortality for delays measured in 24-hour increments. Both published studies [8,9], however, included a majority of episodes that were not ICU-acquired among whom crude hospital mortality rates were about 30%; whereas in our cohort, all episodes were ICU-acquired and the crude mortality rate was 56%. Thus, among more critically-ill patient cohorts, it is possible that any relation between antifungal initiation and outcome may be either confounded (as antifungal therapy is more likely to be initiated earlier in patients with greater disease acuity than in less ill patients) or masked (given that the severity of underlying disease acuity may be the principal predictor of mortality rather than candidaemia or the timing of its treatment).

Optimisation of antifungal regimens represents another potentially important influence on outcome. Several observational studies have defined target pharmacodynamic parameters for fluconazole; including an area under the curve:MIC ratio of 55 and weight normalised dose/MIC ratio of 12, with increased mortality for regimens below these targets [7,10]. However, among our cohort of fluconazole-treated patients, there was no association between outcome and MIC, dose or dose:MIC ratio. Although we were able to adjust fluconazole doses for renal impairment (based on serum creatinine measurements at days 1 and 5), we were not able to adjust for body weight. As above, case mix and severity of illness differences are likely to also be important: in contrast to our cohort, the previous published study cohorts included a minority of ICU patients and overall mortality rates were low (19 to 28%) [7,10]. Taken together, our findings indicate that while optimisation of the timing and dosing of antifungal regimens is clearly an important goal, they may only provide an outcome benefit to patients with moderate illness severity. Conversely, among critically ill patients, even early optimised antifungal regimens after clinical manifestations of candidaemia may not influence outcome. Indeed outcomes might best be improved by antifungal therapy initiation occurring prior to – rather than after – the diagnosis of candidaemia. In this regard, clinical prediction algorithms and more sensitive early diagnostic techniques may assist in guiding early antifungal intervention.

Authoritative guidelines, such as those published by the IDSA [21] and the Australasian Society for Infectious Diseases [22], suggest a number of quality improvement ancillary measures which aim to improve the outcome of candidaemia. These include removal of central venous catheters, follow-up blood cultures and routine ophthalmological examination. Although only limited observational data [23] suggest a clinical and mortality benefit associated with removal of intravascular catheters, it remains generally advocated. In the present study, three-quarters of patients had intravenous catheters removed within five days of candidaemia onset, suggesting that such guidelines are generally but not universally accepted among clinicians. Other advocated strategies were even less frequently adopted: repeat blood cultures to document clearance of candidaemia were performed in only two-thirds of patients; only two-thirds of surviving patients received at least 10 days of antifungal therapy; and an ophthalmological examination was performed in one-third. Of those undergoing ophthalmological examination, 13% had lesions consistent with ocular involvement. Although such lesions may be nonspecific [24], if present, prolongation of antifungal therapy is recommended [21] and vitrectomy with intravitreal antifungal therapy may be required. These findings indicate that despite general support for invasive candidiasis management guidelines, further efforts are required to improve their implementation.

Although this study includes clinical and epidemiological data on a large number of ICU-acquired candidaemia episodes across an entire country, its limitations should be recognised. Illness acuity scores, such as Acute Physiology and Chronic Health Evaluation II scores, were not collected, precluding adjustment of the analyses of prognostic factors associated with candidaemia outcome. However, other markers of illness acuity, such as mechanical ventilation, manifestations of sepsis, renal function and invasive procedures were measured and were included in these analyses. Given that no information on non-candidaemic ICU patients was available, the risk factors for, and attributable consequences of candidaemia among Australian ICU patients remain undefined. Furthermore, we could not accurately determine the incidence of metastatic infective complications associated with candidaemia given the observational nature of the study and the inconsistent performance of ophthalmological, radiological, microbiological and other investigations.

## Conclusions

In summary, this first nationwide study of candidaemia in critically ill ICU patients has provided important information on the epidemiology, clinical management and outcome of ICU-acquired candidaemia. In particular, it suggests that optimisation of the timing and dosing regimens of culture-directed antifungal therapy may not be sufficient to yield improvements in clinical outcome among critically ill ICU patients; rather empirical or preemptive therapy may be required. Furthermore, implementation of strategies to improve and evaluate adherence to guidelines is essential.

## Key messages

• The outcomes of ICU-acquired candidaemia remains poor, with a crude mortality of 56%.

• Among treated patients, host factors, rather than organism-related, pharmacokinetic-related or pharmacodynamic-related factors, are associated with mortality.

• The timing and role of early antifungal therapy in critically-ill ICU patients requires further assessment.

• Strategies are required to improve the implementation of recently published antifungal guidelines.

## Abbreviations

ICU: intensive care unit; IDSA: Infectious Diseases Society of America; IQR: interquartile range; MIC: minimum inhibitory concentration.

## Competing interests

EGP and TCS declare advisory board membership and receipt of research grant support from Pfizer. All other authors declare that they have no competing interests.

## Authors' contributions

DM, TCS, SC, DE and MS conceived, acquired funding, and participated in the design and coordination of the study. EGP participated in the design and coordination of the study. DM and EGP performed the data analysis, were responsible for interpretation of the results and drafted the manuscript. QN managed the study and participated in the data analysis. DE performed species confirmation and antifungal susceptibility testing of all *Candida *isolates. All authors read the manuscript for intellectual content and accuracy and approved the final version.

## Authors' information

Members of the Australian Candidaemia Study included:

Queensland: Cairns Base Hospital (J. McBride); Calboolture Hospital (C. Coulter); Mater Adult Hospital (J. McCormack, K. Walmsley); Princess Alexandra Hospital (D. Looke, B. Johnson, G. Nimmo, G. Playford); Queensland Medical Laboratories (D. Drummond); Rockhampton Hospital (E. Preston); Royal Brisbane Hospital (A. Allworth, J. Faoagali); Sullivan and Nicolaides Pathology (J. Botes, J. Robson); Townsville Hospital (R. Norton); The Prince Charles Hospital (C. Coulter).

New South Wales: Albury Base Hospital (D. Robb); Concord Hospital (T. Gottlieb); Douglass Hanly Moir Pathology (I. Chambers); Gosford Hospital (D. DeWit); Hunter Area Pathology service (J. Ferguson, L. Tierney); Liverpool Hospital (F. Jozwiak, R. Munro); Manning Base Hospital (R. Pickles); Mayne Health (J. Holland); Narrabri District Hospital (F. Groenwald); New Children's Hospital (K. Hale); Orange Base Hospital (R. Vaz);Prince of Wales Hospital (R. Hardiman, C. Baleriola); Royal North Shore Hospital (R. Pritchard, K. Weeks); Royal Prince Alfred Hospital (R. Benn, N. Adams); St George Hospital (R. Lawrence, P. Taylor); St Vincent's Private, and St. Vincent's Public Hospital (J. Harkness, D. Marriott, Q. Nguyen); Sydney Children's Hospital (P. Palasanthrian); Sydney Adventist Hospital (R. Grant); Westmead Hospital (S. Chen, C. Halliday, OC Lee, T. Sorrell); Wollongong Hospital (P. Newton, N. Dennis).

Victoria: Alfred Hosptial (C. Franklin, O. Morrisey, M. Slavin, D. Spelman); Austin and Repatriation Hospital (B. Speed); Bendigo Health Care Group (J. Hellsten, Russell); Melbourne Pathology (S. Coloe); Melbourne Private Hospital (A. Sherman); Monash Medical Centre (T. Korman); PathCare Consulting Pathologists (S. Graves); Peter MacCallum Cancer Institute (M. Slavin, M. Huysmans); Royal Melbourne Hospital (M. Slavin, A. Sherman). South Australia: Flinders Medical Centre (D. Gordon); Royal Adelaide Hospital (K. Rowlands, D. Shaw, W. Ferguson); Women's and Children's Hospital (D. Ellis, R. Handke, S. Davis).

Western Australia: Fremantle Hospital (M. Beaman, J. McCarthy); Royal Perth Hospital (C. Heath); Sir Charles Gairdner Hospital (S. Altmann, I. Arthur, D. Speers).

Tasmania: Launceston General (E. Cox); Royal Hobart Hospital (L. Cooley, A. McGregor).

Northern Territory: Royal Darwin Hospital (B. Currie, G. Lum, D. Fisher). ACT: The Canberra Hospital (P. Collignon, A. Watson).
